# The DNA methylation landscape of musculoskeletal sarcomas

**DOI:** 10.37349/etat.2025.1002319

**Published:** 2025-05-26

**Authors:** Mariana Chantre-Justino, Walter Meohas

**Affiliations:** IRCCS Istituto Romagnolo per lo Studio dei Tumori (IRST) “Dino Amadori”, Italy; ^1^Research Division, National Institute of Traumatology and Orthopaedics, Rio de Janeiro 20940-070, Brazil; ^2^Specialized Care Center for Orthopedic Oncology, National Institute of Traumatology and Orthopaedics, Rio de Janeiro 20940-070, Brazil

**Keywords:** Musculoskeletal sarcomas, bone sarcomas, soft tissue sarcomas, DNA methylation, cell-free DNA, epigenetic therapies

## Abstract

Musculoskeletal sarcomas represent heterogeneous and rare malignant bone and soft tissue tumors, affecting children and adults. Patients exhibiting poor clinical outcomes are often described, being associated with non-response to chemotherapy, amputation needs, or metastatic disease. Potential biomarkers contributing to diagnosis, prognosis, and treatment response could improve this scenario. Despite this, little is known about the genomic aspects of musculoskeletal sarcomas. DNA methylation is the most studied epigenetic mechanism, where changes in methylation profiling are characteristic hallmarks of cancer. Cancer-related methylome profiling has been investigated both in tumor biopsies (genomic DNA) and liquid biopsies (cell-free DNA). Epigenetic therapies by using DNA-demethylating drugs are promising strategies for cancer treatment. This review will discuss translational studies describing how DNA methylation landscape of musculoskeletal sarcomas can be a powerful molecular tool to improve diagnostic accuracy, predict prognosis, and treatment response. Additionally, this review will describe the promising role of epigenetics-targeted drugs as well as the ongoing clinical trials for sarcomas, highlighting the challenges and future directions.

## Introduction

Musculoskeletal sarcomas are a heterogeneous and clinically complex group of rare malignant neoplasms of mesenchymal origin that arise in bones and soft tissues, accounting for about 1% of cancers [1–4]. These sarcomas can affect both children and adults, and the presence of metastatic disease, most commonly in the lungs, is associated with poor prognosis [1, 2]. Osteosarcoma (OS), Ewing sarcoma (ES), and chondrosarcoma (CS) are the most common types of malignant bone tumors, whereas liposarcoma (LPS) and synovial sarcoma (SS) are the most common types of malignant soft tissue tumors [1–3]. Treatment strategies for musculoskeletal sarcomas generally include surgery and chemotherapy [5, 6]. Despite treatment efforts, the management of sarcomas remains challenging, and poor clinical outcomes are frequently described. Since treatment options for sarcomas have remained limited over decades, novel therapeutic strategies are needed to address this challenge. Advances in understanding cancer genomics have substantially impacted diagnostic and therapeutic evaluations for precision medicine. Therefore, new molecular information about the biology of musculoskeletal sarcomas could better describe tumor heterogeneity and reveal candidate genes clinically relevant to improve specific therapies. In this review, we will discuss the clinical implications of changes in methylation profiling for musculoskeletal sarcomas, highlighting the emerging role of DNA methylation-based classifiers to refine the diagnosis, as well as the novel targeted therapies and future directions.

## Common genomic alterations in musculoskeletal sarcomas

Musculoskeletal sarcomas are tumor entities harboring considerable genomic heterogeneity, with point mutations, gene fusions, and gene amplifications being the most well-documented genetic alterations. The *TP53* and *RB1* tumor suppressor genes are frequently mutated in OS [7, 8]. The major genetic driver event in ES tumors is the oncogenic fusion *EWSR1*::*FLI1* from the translocation t(11;22)(q24;q12) [9, 10]. CS tumors commonly harbor mutations in the isocitrate dehydrogenase 1 and 2 genes (*IDH1*/*IDH2*), which can predict clinical outcomes [11, 12]. For soft tissue sarcomas, *MDM2*/*CDK4* amplification is a common molecular finding described in well-differentiated and dedifferentiated LPS, whereas the *FUS*::*DDIT3* fusion gene from the translocation t(12;16)(q13;p11) is characteristic of myxoid LPS [13, 14]. The *SS18*::*SSX* fusion gene from the translocation t(X;18)(p11.2;q11.2) is commonly identified in SS [15]. In childhood cancer, rhabdomyosarcoma (RMS) is the most common pediatric soft tissue sarcoma that has been classified into four histological subtypes, in which the alveolar RMS (ARMS) subtype commonly harbor the *PAX*::*FOXO1* fusion gene [*PAX3*::*FOXO1* from t(2;13)(q35;q14); *PAX7*::*FOXO1* from t(1;13)(p36;q14)] and the embryonal RMS (ERMS) subtype is characterized by loss of heterozygosity (LOH) at the 11p15 locus [16, 17]. These main genetic hallmarks described in musculoskeletal sarcomas are listed in Table 1.

**Table 1:** Summary of main genetic alterations identified in musculoskeletal sarcomas described in this review

**Sarcoma subtype**	**Main genetic hallmarks**	**Genes/location**	**References**
OS	Mutation	*TP53* and *RB1*	[7, 8]
ES	Translocation t(11;22)(q24;q12)	*EWSR1*::*FLI1*	[9, 10]
CS	Mutation	*IDH1* and *IDH2*	[11, 12]
WDLPS; DDLPS	Amplification	*MDM2* and *CDK4*	[13, 14]
MLPS	Translocation t(12;16)(q13;p11)	*FUS*::*DDIT3*	[13, 14]
SS	Translocation t(X;18)(p11.2;q11.2)	*SS18*::*SSX*	[15]
Alveolar RMS	Translocations: t(2;13)(q35;q14); t(1;13)(p36;q14)	*PAX3*::*FOXO1*; *PAX7*::*FOXO1*	[16, 17]
Embryonal RMS	LOH	11p15 locus	[16, 17]

OS: osteosarcoma; ES: Ewing sarcoma; CS: chondrosarcoma; *IDH1*: isocitrate dehydrogenase 1 gene; WDLPS: well-differentiated liposarcoma; DDLPS: dedifferentiated liposarcoma; MLPS: myxoid liposarcoma; SS: synovial sarcoma; RMS: rhabdomyosarcoma; LOH: loss of heterozygosity

**Figure 1:**
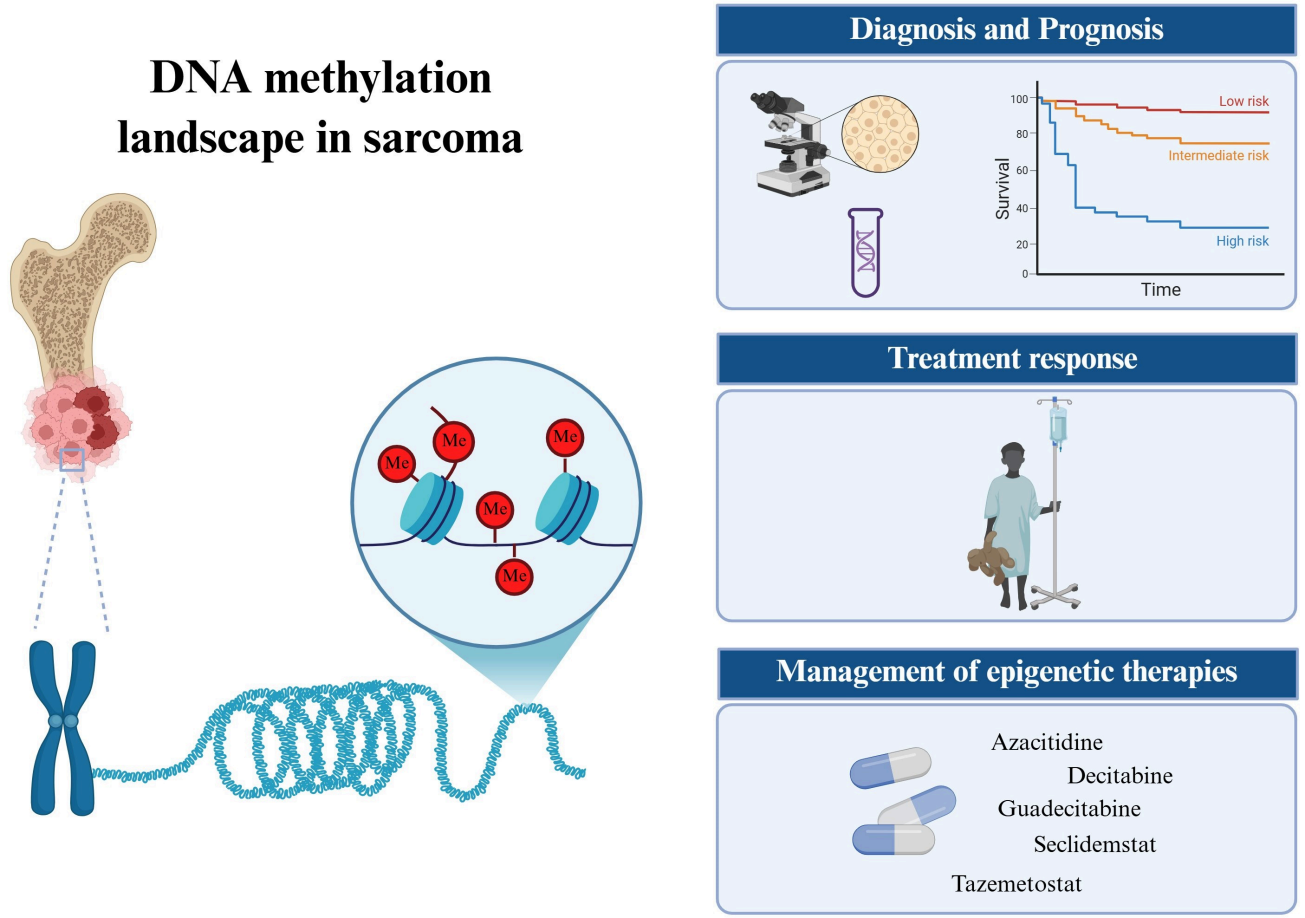
**Representative illustration of DNA methylation landscape in musculoskeletal sarcomas and its clinical applications.** Epigenetic changes by DNA methylation in promoter regions of CpG islands and/or histone methylation can facilitate the development and progression of sarcomas. In addition to providing a more in-depth knowledge of tumorigenesis, the methylome profiling in sarcomas can be investigated for different clinical purposes: refining the diagnosis and predicting the prognosis, assessing variations in treatment response, and assisting in the management of epigenetics-targeted drugs. Epigenetic therapies by using DNA-demethylating drugs are promising strategies for cancer treatment and some clinical trial studies, including pediatric and adult patients with bone and soft tissue sarcomas, are under investigation. Me: methylation (CpG islands or histones). Created in BioRender. Justino, M. (2025) https://BioRender.com/lrsdqyc

In addition to genetic alterations, increasing evidence points to other molecular events involved in cancer biology [18]. Therefore, the biological and clinical complexity of musculoskeletal sarcomas suggests that additional molecular alterations impact these neoplasms. Epigenetic mechanisms are reversible molecular events influencing changes in chromatin structure that result in transcriptional regulation, which can lead to inactivation of critical genes followed by increased genomic instability [19]. Therefore, epigenetic changes are relevant genomic events for describing the clinical complexity of most tumors. Epigenetic changes can be regulated by DNA methylation, histone modifications, and non-coding RNAs (ncRNAs), where DNA methylation is the most studied epigenetic inactivation mechanism in cancer investigations [19–21]. The DNA methylation mechanism is catalyzed by DNA methyltransferases (DNMTs), which are responsible for the addition of a methyl group (CH3) in the 5' carbon of the cytosine on the CpG islands in the promoter region, resulting in chromatin compaction and, consequently, transcriptional inactivation [22]. Cancer cells often exhibit global DNA hypomethylation and specific hypermethylation at CpG sites [23]. Therefore, cancer-related methylome profiling can be used to complement histopathological analysis and genetic testing to refine diagnosis, predict prognosis, assess treatment efficacy, and assist in the management of targeted therapies (Figure 1).

## DNA methylation profiling in musculoskeletal sarcomas: associations with tumor development and progression

The investigation of epigenetic signatures through DNA methylation profiling in musculoskeletal sarcomas can be a complementary assessment to describe the clinical complexity and genomic heterogeneity of these tumors. By genome-wide DNA methylation, Tian et al. [24] described an overall hypomethylation (~76.7%) of CpG dinucleotides and several hypermethylations of some tumor suppressor gene promoters in OS samples, contributing to chromosome instability and OS development. Rosenblum et al. [25] reported an association between increased genome-wide DNA methylation and disease recurrence in pediatric patients with OS. Furthermore, the authors identified greater levels of DNA methylation at loci within gene enhancers, gene bodies, and intergenic regions. Park et al. [26] conducted a microarray-based DNA methylation study in patients with ES. The authors identified 92 genes that were significantly hypermethylated, in which the overall methylation mean was significantly greater in patients who did not survive compared to surviving patients. Sheffield et al. [27] identified substantial DNA methylation heterogeneity in ES samples, where DNA hypomethylation was observed in binding sites and correlated enhancers of the oncogenic fusion protein EWS::FLI1. It has been reported that the histone methyltransferase activity of enhancer of zeste homolog 2 (EZH2) via trimethylation of H3K27 (H3K27me3) in ES tumors can be directly mediated by the EWS::FLI1 through binding to the *EZH2* promoter region, resulting in EZH2 expression [28]. CS tumors commonly harbor *IDH1*/*IDH2* mutations, resulting in high levels of the oncometabolite 2-hydroxyglutarate (2-HG). It has been reported that elevated levels of 2-HG affect DNA methylation and histone methylation, in which the DNA hypermethylation observed in mesenchymal stromal cells could induce the CS development [29, 30]. These findings are in accordance with a study of Nicolle et al. [31], which identified a genome-wide DNA hypermethylation in CS tumors exhibiting *IDH* mutations.

Distinct DNA methylation signatures were identified between fusion-positive (*PAX*::*FOXO1*) and fusion-negative RMS, with fusion-positive tumors exhibiting lower overall methylation levels compared to fusion-negative tumors [32]. Similarly, Tombolan et al. [33] reported distinct methylation profiling between fusion-positive and fusion-negative RMS. Additionally, the authors demonstrated that metastatic and non-metastatic RMS also display distinct DNA methylation patterns, with hypermethylation of several protocadherin genes, particularly the *PCDHA4* promoter region, associated with metastatic disease. A study conducted by Liu et al. [34] investigated the influence of the DNA methylation status from the guanine nucleotide exchange factor T (GEFT) on epithelial-mesenchymal transition (EMT) or mesenchymal-epithelial transition (MET) in RMS progression. By MALDI-TOF MS technique, the authors observed *GEFT* promoter hypomethylation in RMS compared to normal tissues, leading to GEFT protein overexpression and promoting tumor invasion and metastasis.

Tumor suppressor genes play a key antiproliferative role in maintaining genomic stability. The mutational or epigenetic inactivation of several tumor suppressor genes, leading to loss of function, is a critical event associated with tumor initiation and progression [35]. Oh et al. [36] evaluated tumor samples from patients with OS and reported DNA hypermethylation of the *p16^INK4A^* and *p14^ARF^* genes in 16% and 47% of the samples, respectively. Additionally, *p14^ARF^* methylation was related to poor survival. Röpke et al. [37] investigated the DNA methylation status of 8 tumor suppressor genes in a dedifferentiated CS case and found *p16^INK4A^* and *E-cadherin* promoter hypermethylation in both dedifferentiated CS sites, whereas methylation of the *FHIT* promoter was found only in the highly malignant dedifferentiated tumor component. Jin et al. [38] observed *RUNX3* promoter hypermethylation in tumor tissue samples from patients with CS, which resulted in decreased *RUNX3* mRNA levels and was correlated with a poor prognosis. RMS tumors harboring *PAX3*::*FOXO1* fusion gene showed promoter hypermethylation compared to *PAX7*::*FOXO1* positive tumors [39]. Hou et al. [40] observed significant DNA hypermethylation in several genes, including the tumor suppressor genes *CDKN2A* and *RASSF1A*, in tumor tissues compared to normal tissues from patients with OS, suggesting that DNA hypermethylation of multiple genes may contribute to OS development. The authors also reported a significant difference in DNA methylation levels between patients with metastatic and nonmetastatic OS.

## DNA methylation-based classification of musculoskeletal sarcomas

The DNA methylation signature has emerged as a promising molecular tool to refine the diagnosis of musculoskeletal sarcomas. Wu et al. [41] developed a DNA methylation-based classifier and observed distinct methylation profiling between OS, ES, and SS, which can aid diagnosis when standard techniques are inconclusive. By array-based DNA methylation, Koelsche et al. [42] reported that DNA methylation profiling is highly useful as a diagnostic tool, as it precisely assigned to specific sarcoma subtypes some tumors with previously unsatisfactory status of “Ewing-like” sarcoma and small blue round cell tumors not otherwise specified. Lyskjær et al. [43] investigated 820 sarcoma samples by a methylation-based classifier and observed a prediction in 61% of cases, in which the histological diagnosis had concordant findings with the predicted methylation class in 88% of cases. Additionally, the classifier performed best in the diagnosis of mesenchymal CS (88% sensitivity), whereas lower classification and accuracy rates were found among sarcoma subtypes, genomically complex and with high tumor heterogeneity, such as pleomorphic LPS (29% sensitivity). Roohani et al. [44] reported that the methylation-based classifier for sarcomas confirmed the diagnosis or suggested a novel diagnostic category for the patient. Barenboim et al. [45] developed a methylation-based classifier to detect the BRCAness status in OS samples. BRCAness refers to samples harboring defects in genes from homologous recombination repair (HRR) and resembling many features of *BRCA*-mutant tumors. In Barenboim’s work, the BRCAness-positive group exhibited lower DNA methylation signal compared to the BRCAness-negative group, suggesting upregulation of gene expression in the BRCAness-positive group. The authors identified 449 upregulated and 1,079 downregulated genes in the BRCAness-positive group, including genes involved in DNA replication, DNA repair, and cell cycle regulation. These data on BRCAness status in OS could contribute to the decision to administer poly ADP-ribose polymerase inhibitors (PARPis).

## DNA methylation status and response to treatment

The DNA methylation status may be useful for predicting treatment response. By genome-wide DNA methylation analysis, Lietz et al. [46] reported that patients with OS in the hypomethylated group responded better to standard chemotherapy (methotrexate, doxorubicin, and cisplatin) and exhibited better survival rates than did those in the hypermethylated group. Regarding immunotherapy response, Starzer et al. [47] evaluated 27 soft tissue sarcoma samples and 8 OS samples and identified two main methylation clusters between responders and non-responders to anti-PD-1 immunotherapy (pembrolizumab or nivolumab), regardless of the sarcoma subtype. Thus, these findings of differential methylation could serve as predictors of the immunotherapy response in patients with sarcoma. The O^6^-methylguanine-DNMT (*MGMT*) gene encodes a DNA repair enzyme that removes alkylating agents, which may influence the chemotherapy response. In patients with glioblastoma, the methylation status of the *MGMT* promoter is used to predict the treatment response to temozolomide (TMZ), an alkylating agent [48]. Therefore, epigenetic inactivation of *MGMT* by DNA methylation is an important criterion for evaluation of therapeutic response in cancer patients and could be further investigated in musculoskeletal sarcomas. Salah et al. [49] reported that 25% (5/20) of patients with advanced ES had *MGMT* promoter methylation; however, no significant correlation was found between the *MGMT* methylation status and clinical outcomes following salvage irinotecan and TMZ chemotherapy regimens. Interestingly, the authors reported that the median progression-free survival (PFS) was significantly longer in patients with methylated *MGMT* following the standard primary protocol (vincristine, doxorubicin, and cyclophosphamide alternating with ifosfamide and etoposide), with a PFS of 27.8 months for methylated *MGMT* and 8.6 months for those with unmethylated *MGMT*. Cisplatin is another important alkylating agent commonly used for the treatment of cancers, including sarcomas. Cui et al. [50] reported that patients with OS and detectable methylation of the *MGMT* gene promoter had a higher tumor necrosis rate after chemotherapy (cisplatin, adriamycin, and ifosfamide) and therefore a better treatment effect than patients exhibiting unmethylated *MGMT* gene promoter.

## Circulating biomarkers: cell-free DNA methylation signatures in musculoskeletal sarcomas

Liquid biopsy is an emerging area of investigation based on minimally invasive procedures to track potential tumor-related molecular alterations through biological fluids, thus providing additional information on tumor dynamics and heterogeneity [51]. Therefore, the liquid biopsy approach is especially attractive for diagnosing and monitoring solid tumors that require invasive biopsies. The cell-free DNA (cfDNA) is the most investigated category in liquid biopsy, in which differences in amounts and genomic signatures can be observed between physiological and pathological conditions [52]. Furthermore, cfDNA fragmentation patterns could add diagnostic and prognostic value [53]. Despite it being an attractive approach in oncology, there are few studies on liquid biopsy investigations in patients with musculoskeletal sarcoma. Udomruk et al. [54] reported that the size of cfDNA fragments was significantly shorter in patients with OS than in healthy donors. Additionally, the authors reported that short cfDNA fragments were a prognostic predictor and a major source of mutations.

**Figure 2:**
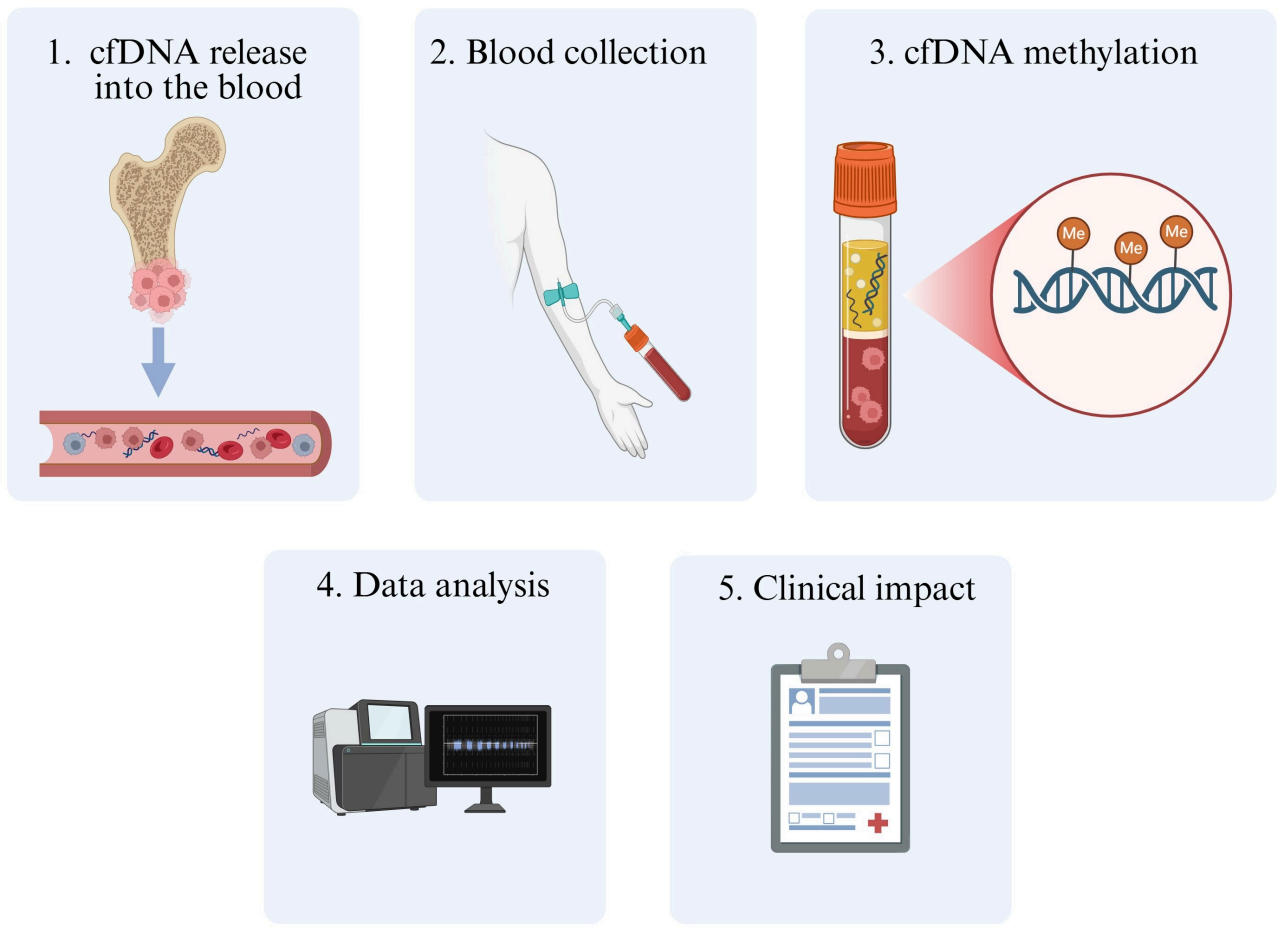
**Representative illustration of cfDNA methylation investigations in musculoskeletal sarcomas.** Firstly, cfDNA is released into biofluids, usually being derived from processes of cell death (e.g., apoptosis, necrosis, and autophagy). After blood collection, cfDNA can be isolated to detect methylation signatures through different methodological approaches, unraveling their clinical impact. Me: methylation; cfDNA: cell-free DNA. Created in BioRender. Justino, M. (2025) https://BioRender.com/fwq7ula

DNA methylation signatures can also be detected in cfDNA from different tumors. Peneder et al. [55] reported that pediatric patients with ES exhibited high levels of shorter cfDNA fragments compared to healthy controls. The authors also identified corresponding DNA methylation profiles between cfDNA and tumor biopsies. Lyskjær et al. [56] observed that the detection of cfDNA methylation or high levels of cfDNA preoperatively was correlated with the lowest survival rates in patients with OS. Despite these few aforementioned studies, methylation-based cfDNA analyses should be encouraged for musculoskeletal sarcomas to better characterize the methylome profiling and to unveil novel biomarkers for this disease (Figure 2).

## Epigenetic therapies for musculoskeletal sarcomas

The treatment of soft tissue and bone sarcomas remains challenging, as most patients do not respond effectively to therapy and exhibit poor outcomes. Therefore, novel and promising treatment strategies are needed. Currently, several epigenetic drugs have been approved by the Food and Drug Administration (FDA) for cancer treatment [57]. Blockade of aberrant DNA hypermethylation using pharmacological inhibitors can be applied in tumors with known dysregulation of DNA methylation signatures. Thus, epigenetic treatment involving the use of DNA-demethylating drugs to reverse tumor suppressor functions can also be a therapeutic strategy for patients with musculoskeletal sarcomas. The azacitidine and decitabine are hypomethylating agents with known DNMT inhibitor (DNMTi) activities [57, 58].

An in vitro study showed that decitabine facilitated the immune recognition in pediatric sarcoma cell lines [59]. Numoto et al. [60] investigated 74 soft tissue sarcoma samples and observed *RASSF1A* methylation in 47.6% of the SS samples and in 18.9% of the other soft tissue sarcomas. Additionally, the authors observed demethylation of *RASSF1A* and increased *RASSF1A* mRNA levels in SS cell lines treated with 5-aza-2'-deoxycytidine (decitabine). Gutierrez et al. [61] reported that low-dose decitabine in combination with gemcitabine significantly improved survival and slowed tumor growth in a mouse model of high-grade sarcoma. Higuchi et al. [62] reported that oral administration of recombinant methioninase and decitabine resulted in tumor growth arrest in an undifferentiated soft-tissue sarcoma patient-derived orthotopic xenograft mouse model.

Some clinical trial studies involving epigenetic agents through DNA-demethylating drugs are under investigation for musculoskeletal sarcomas. A phase Ib clinical trial (NCT02959164) in advanced soft tissue and bone sarcomas reported that low-dose decitabine combined with fixed-dose infusion of gemcitabine was moderately toxic [63]. In another phase I clinical trial (NCT01241162), the use of decitabine followed by a dendritic cell vaccine for children with relapsed/refractory solid tumors, including ES, OS, and RMS, was feasible and well tolerated in some cases [64]. A phase 2 clinical trial (NCT04340843) in patients with advanced conventional CS is evaluating a combination regimen with guadecitabine, a DNMTi, and belinostat, a histone deacetylase inhibitor [65]. However, the authors reported that this study is currently on hold, pending completion of the safety lead-in. Lysine-specific demethylase 1 (LSD1) specifically demethylates histones H3K4me1/2 and H3K9me1/2, resulting in transcriptional repression. LSD1 overexpression has been associated with cancer development and progression; thus, LSD1 inhibitors are under investigation [57, 66]. Seclidemstat (SP-2577) is an oral and selective LSD1 inhibitor, for which a phase I/II clinical trial (NCT03600649) is currently active for patients ≥ 12 years old with ES and relapsed/refractory disease [67]. Tazemetostat is an FDA-approved EZH2 inhibitor for patients harboring wild-type or mutant *EZH2* [57, 68]. EZH2 dysregulation can be caused by upregulation or mutations that increase its methyltransferase activity, resulting in H3K27me3 and gene silencing. Patients with ES harboring activating *EZH2* mutations have been associated with an aggressive phenotype and thus could benefit from tazemetostat treatment [69]. A phase 2 clinical trial (NCT03213665) evaluated tazemetostat in pediatric patients with relapsed or refractory disease and found that *EZH2* mutations were present in 3/20 (15%) tumors, 2/20 of which were identified in patients with ES [70]. The study revealed that tazemetostat prolonged stable disease (> 6 months) in 33% of patients. The currently available and developing epigenetic therapies for musculoskeletal sarcomas described in this review are summarized in Table 2.

**Table 2:** Epigenetic therapies for musculoskeletal sarcomas discussed in this review

**Sarcoma subtype**	**Drug**	**Target**	**ClinicalTrials.gov identifier**	**Ref.**
Mixed groups	Decitabine	DNMTi	NA	[59]
SS	Decitabine	DNMTi	NA	[60]
UPS	Decitabine plus gemcitabine	DNMTi	NA	[61]
USTS	Decitabine plus methioninase	DNMTi	NA	[62]
Mixed groups	Decitabine plus gemcitabine	DNMTi	NCT02959164	[63]
Mixed groups	Decitabine plus dendritic cell vaccine	DNMTi	NCT01241162	[64]
CS	Guadecitabine plus belinostat	DNMTi; HDACi	NCT04340843	[65]
ES	Seclidemstat (SP-2577)	LSD1 inhibitor	NCT03600649	[67]
Mixed groups	Tazemetostat	EZH2 inhibitor	NCT03213665	[70]

NA: not applicable; DNMTi: DNA methyltransferase inhibitor; SS: synovial sarcoma; UPS: undifferentiated pleomorphic sarcoma; USTS: undifferentiated-soft tissue sarcoma; CS: chondrosarcoma; HDACi: histone deacetylase inhibitor; ES: Ewing sarcoma; LSD1: lysine-specific demethylase 1; EZH2: enhancer of zeste homolog 2

## Challenges and future directions

The molecular characterization of musculoskeletal sarcomas remains challenging, especially for soft tissue and bone sarcomas that do not exhibit known genomic hallmarks, such as point mutations or gene fusions. Importantly, studies involving DNA methylation in soft tissue sarcomas are far more limited; thus, further investigations are needed. Additionally, pediatric and adult malignant neoplasms are characterized by distinct genetic drivers, in which adult patients carry a high number of somatic mutations, whereas pediatric patients are characterized by germline alterations and lower mutational burden [71]. Similarly, distinct epigenetic markers and differentially methylated regions may predict the risk of childhood and adulthood cancers [72–74]. Since epigenetic markers are not yet well established for sarcomas, comparing the methylome profiles of pediatric and adult patients may provide valuable insights.

DNA methylation-based approaches, such as array-based platforms and methylation sequencing, have been extensively used to assess the methylome profiling of sarcomas, demonstrating their relevance for translational medicine and future diagnostic applications [27, 42]. In addition, liquid biopsy through blood-based cfDNA methylation signatures could also be an effective investigation strategy for musculoskeletal sarcomas, as it provides additional information on tumor dynamics and heterogeneity. Finally, epigenetic therapies involving the use of DNA-demethylating drugs are promising strategies for cancer treatment. To date, there are few clinical trials involving DNA-demethylating drugs for musculoskeletal sarcomas, and these studies are still in the early stages. Despite these challenges, further methylome studies should be encouraged to identify potential molecular targets to improve patient outcomes.

## Conclusions

DNA methylation signatures could better describe the clinical complexity of musculoskeletal sarcomas. The DNA methylation landscape of patients with bone and soft tissue sarcomas could provide additional genomic information to refine diagnosis, prognosis, and therapeutic intervention.

## Abbreviations

cfDNA: cell-free DNA
CS: chondrosarcoma
DNMTs: DNA methyltransferases
ES: Ewing sarcoma
EZH2: enhancer of zeste homolog 2
GEFT: guanine nucleotide exchange factor T
*IDH1*: isocitrate dehydrogenase 1 gene
LPS: liposarcoma
LSD1: lysine-specific demethylase 1
*MGMT*: O^6^-methylguanine-DNA methyltransferase
OS: osteosarcoma
RMS: rhabdomyosarcoma
SS: synovial sarcoma

## Declarations

### Copyright

© The Author(s) 2025.
